# A Systematic Framework for Analyzing Observation Data in Patient-Centered Registries: Case Study for Patients With Depression

**DOI:** 10.2196/18366

**Published:** 2020-10-29

**Authors:** Maryam Zolnoori, Mark D Williams, William B Leasure, Kurt B Angstman, Che Ngufor

**Affiliations:** 1 Mayo Clinic Rochester, MN United States

**Keywords:** patient-centered registry, collaborative care model, care coordination management, integrated behavior health, systematic framework

## Abstract

**Background:**

Patient-centered registries are essential in population-based clinical care for patient identification and monitoring of outcomes. Although registry data may be used in real time for patient care, the same data may further be used for secondary analysis to assess disease burden, evaluation of disease management and health care services, and research. The design of a registry has major implications for the ability to effectively use these clinical data in research.

**Objective:**

This study aims to develop a systematic framework to address the data and methodological issues involved in analyzing data in clinically designed patient-centered registries.

**Methods:**

The systematic framework was composed of 3 major components: visualizing the multifaceted and heterogeneous patient-centered registries using a data flow diagram, assessing and managing data quality issues, and identifying patient cohorts for addressing specific research questions.

**Results:**

Using a clinical registry designed as a part of a collaborative care program for adults with depression at Mayo Clinic, we were able to demonstrate the impact of the proposed framework on data integrity. By following the data cleaning and refining procedures of the framework, we were able to generate high-quality data that were available for research questions about the coordination and management of depression in a primary care setting. We describe the steps involved in converting clinically collected data into a viable research data set using registry cohorts of depressed adults to assess the impact on high-cost service use.

**Conclusions:**

The systematic framework discussed in this study sheds light on the existing inconsistency and data quality issues in patient-centered registries. This study provided a step-by-step procedure for addressing these challenges and for generating high-quality data for both quality improvement and research that may enhance care and outcomes for patients.

**International Registered Report Identifier (IRRID):**

DERR1-10.2196/18366

## Introduction

### Background

Patient-centered registries can complement standard electronic health records (EHRs) for the purpose of coordinating an organized response to a subgroup of patients with the goal of improving health care quality and value [1]. The National Institute of Health defines a clinical registry as “a collection of information about individuals, usually focused around a specific diagnosis or condition” [2]. Properly designed and executed registries can play essential roles in enabling patient-centered care, assessing disease burden, evaluating disease management and health care services, disseminating and using information about targeted diseases and health services, and conducting comparative effectiveness outcomes research [3-5].

The EHR has revolutionized the capture and storage of clinical data. However, as patients often seek care across multiple health systems, use multiple pharmacies, and change insurance carriers, the data captured in EHRs may be limited to better characterize patient cohorts or evaluate longitudinal clinical care [3]. To enable a comprehensive view of a patient and enable research that can guide policy and best practices, there is a need for patient-centered registries to be integrated with the EHR, administrative claims data, and pharmacy databases [6]. Additionally, there is a need for registry oversight to ensure data integrity and the conceptual and methodological frameworks for generating and evaluating data-driven hypotheses. In this study, the patient-centered registries primarily consisted of patients’ clinical outcomes, self-management ratings, and measures of satisfaction with care.

Mental health disorders are common (46.6 million of the US population in 2017) [7], create significant disability and losses in productivity [8], and lead to substantial health costs. The majority of patients in the United States, however, do not receive effective mental health care [9]. The majority of patients with mental health problems present in primary care settings [10], where collaborative care models (CCMs) have been tested and implemented with positive outcomes in over 75 randomized controlled trials [11]. CCMs for mental health problems address the lack of access to specialty care with an evidence-based model. CCM has several critical elements that typically include (1) a care coordinator to connect with and manage the patient with a given illness, (2) a method of identification and tracking of these patients using a patient-centered registry, (3) the participation of a specialist providing a regular review of these patients with oversight of the care coordinator, and (4) a primary care provider who continues to care for these patients. Depression in adults is a very common target for CCM, based on the improving mood-promoting access to collaborative treatment (IMPACT) model [12]. New sources of reimbursement for this model from the Center for Medicare and Medicaid Studies provide incentives for clinical practices to adopt collaborative care. Each practice must create its own patient-centered registry for clinical management of those patients in collaborative care, assessing outcomes, and potentially a clinical research.

In 2008, the division of Integrated Behavioral Health (IBH) at the Mayo Clinic began implementing CCM for adult depression in primary care. As a part of that effort, a patient-centered registry was built with the ability to track both clinical outcomes and care engagement for the patient population over time. The registry was designed to support the implementation of CCM to deliver integrated and coordinated treatment for depression [13]. Outcomes and changes in treatments for patients were systematically captured and summarized in a transparent and actionable manner, which promoted more rapid changes in treatment for patients who were not improving compared with practice as usual [14]. The integrated nature of the CCM enabled providers to systematically take into account the complex medical, psychological, social, and cultural factors affecting a patient’s illness and provide personalized treatment plans to ensure that treatment goals were met.

The depression registry included information on diverse patients with respect to disease severity, treatment protocols, comorbidities, and socioeconomic and ethnic backgrounds. The Mayo Clinic system is a multispecialty practice in a city of 100,000 with both primary and specialty care included within the same EHR along with hospital and emergency room data. For this implementation, the capacity has existed to potentially integrate the registry data with administrative, pharmacy, emergency, and hospitalization databases as well as patients’ social determinants of health and personal health records. Despite the robust design and implementation of the depression registry, there were inherent data limitations that impeded effective research. Specifically, as the registry has prevalent data quality issues (data inconsistency, accuracy, and completeness), defining or utilizing longitudinal outcomes for research has been challenging. Additionally, identifying patient cohorts (group of patients sharing similar clinical or utilization characteristics) in the registry for a retrospective study is a challenging process.

### Objectives

Previous studies have discussed the challenges of evaluating data-driven hypotheses using data accumulated in patient-centered registries and have offered general guidelines to address these challenges. For example, Gliklich et al [15] provided an overview of data quality issues including data completeness, missing values, and data accuracy in registries and listed available solutions for the problems. In another study, Kodra et al [16] discussed 6 dimensions of data quality such as data usefulness, accessibility, and timeliness in registries for rare diseases. They also proposed methods for evaluating the quality of data against the 6 dimensions. Although helpful in identifying problems and strategies, these studies did not propose a systematic framework to address the methodological challenges of identifying patient cohorts in the patient registries, specifically in patient-centered registries for mental disorders. For the purpose of this study, we defined the systematic framework as an analytical tool, consisting of structured components that addressed the challenges of evaluating a data-driven hypothesis using accumulated data in a patient-centered registry.

To demonstrate the general applicability of the systematic framework, we applied it to generate an analytic sample of patients from the depression registry and used the sample to describe the structure and characteristics of the implemented CCM.

## Methods

The major components of the systematic framework include (1) development of a data flow diagram (DFD) to visualize components of the registry; (2) data quality assessment, which focused on the analysis of data errors (accuracy) and missingness (completeness); and (3) identification of patient cohorts, which covered the challenges of identifying comparable patient cohorts in longitudinal clinical care. We demonstrated the feasibility and usefulness of this framework using the depression registry. By following the analytical steps of this framework, we produced high-quality data and identified major patient subgroups for subsequent cross-sectional or longitudinal studies. This study was reviewed and approved by the Institution Review Board at the Mayo Clinic. See Multimedia Appendix 1 for more information about the depression registry.

### Patient Registry DFD

In exploring the use of patient-centered registries for research, a DFD [17] provides research teams with a high-level view of the scope and focus of the registry. Additionally, a DFD allows the reader to visualize the interaction between different components of the registry and the clinical decision-making process designed to manage patient care. This visualization subsequently facilitates the process of generating rules for data quality assessment and defining patient cohorts for a specific research question. Designing a DFD for a patient-centered registry should include 3 major parts. First, the eligibility portion of the DFD needs to present information about the criteria and evaluation models for identifying eligible patients in the registry. Second, an enrollment section of a DFD should include information about enrollment, specifically the patients’ decision to enroll in the registry after being approached by clinicians and a list of patients who met eligibility requirements but were not approached. Finally, the third section of a DFD should provide information about the flow of enrolled patients in the registry during and after receiving the intervention. This part can also include information on the process of data collection at the initial and follow-up visits for enrolled patients.

**Figure 1:**
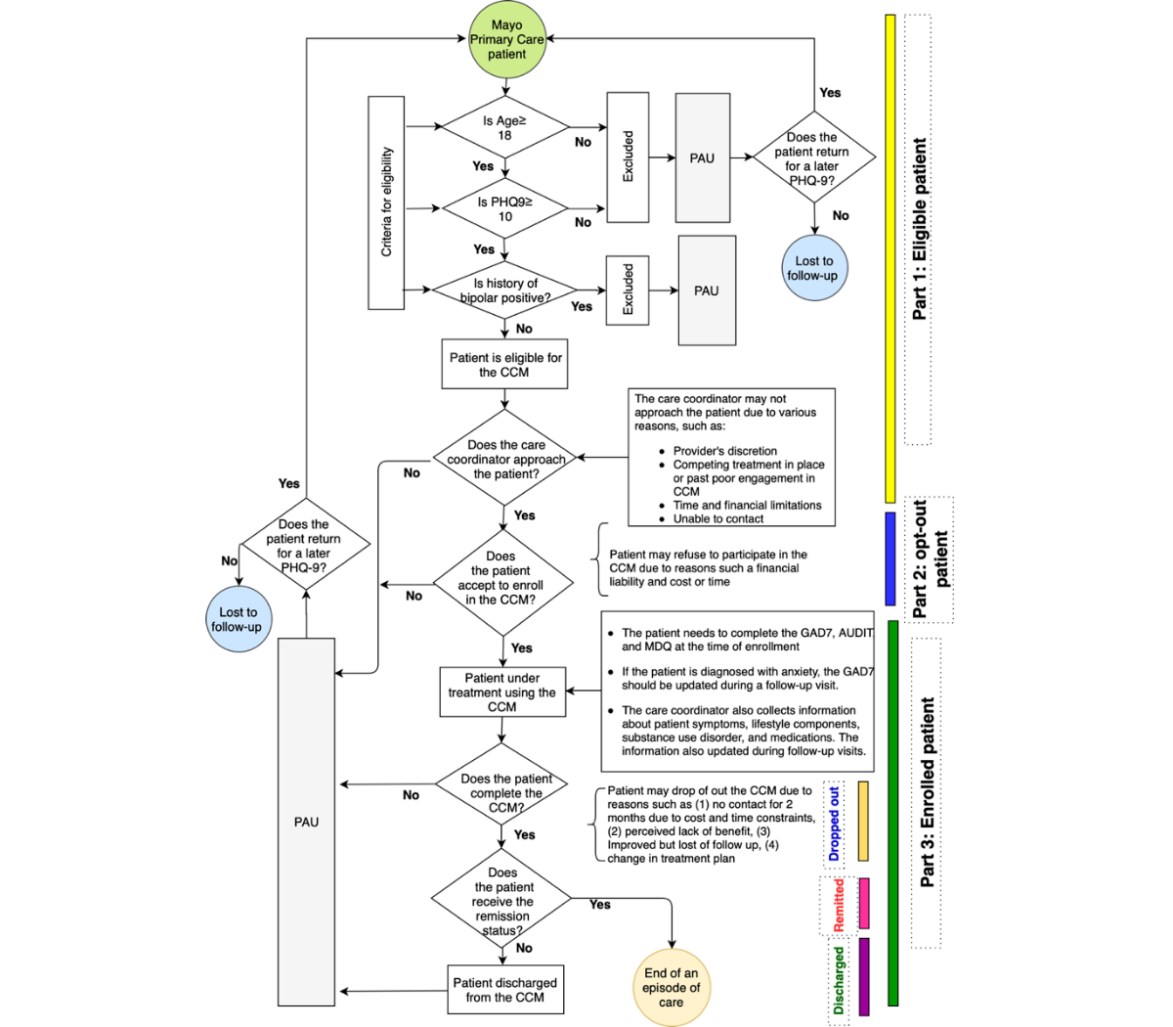
Data flow diagram of the Integrated Behavioral Health registry. AUDIT: Alcohol Use Disorders Identification Test; CCM: Care Coordination Model; GAD7: General Anxiety Disorder-7; MDQ: Mood Disorder Questionnaire; PAU: Practice As Usual.

We used the DFD to visualize components of the depression registry and the CCM intervention (Figure 1). The first part of the depression DFD presents criteria for identifying eligible patients for the CCM intervention, including a clinical diagnosis of depression with a Patient Health Questionnaire (PHQ-9) ≥10 [18], age ≥18 years, and no history of bipolar disorder. The second part of the DFD focused on eligible patients, including those who refused to enroll in the CCM. The patient’s refusal of the intervention was recorded as an *opt-out* status in the depression registry. A patient may refuse to enroll in the CCM for various reasons, including cost or time commitment. Additionally, a patient may not be approached for reasons including lack of space in the program, perceptions that the patient was already involved in analogous services, and past lack of interest in participating. Finally, the last part of the DFD demonstrated that an enrolled patient could experience 3 potential outcomes: *drop out* (stopping the CCM without completing a course of treatment), *remitted* (remission from depression), and *discharged* (discharged from the CCM without reaching the remission status). The enrolled patients were required to complete questionnaires related to anxiety symptoms [19], bipolar disorder [20], and alcohol use disorder [21] and to provide information about lifestyle components and medication use. This information was updated in follow-up visits and was used for assessing the patient’s treatment plan.

Overall, the DFD provided insight into the underlying structure of the data, the main patient cohorts available in the registry, and the potential limitations of retrospective studies based on data from the patient-centered registries. For example, the DFD showed that the depression registry included 3 major patient cohorts: nonapproached, opt out, and enrolled. Additionally, the patient information in the registry consisted of both structured and unstructured data (clinical notes), creating additional variables for interpretation.

### Data Quality Assessment

Identifying the data quality issues and adopting appropriate solutions to solve those issues are a critical part of data processing in patient-centered registries that evolve over time. The research team may use available frameworks of data analysis errors, such as those described by Tallentire et al [22] or Kahn et al [23] to evaluate the quality of observation data in the registry against 6 dimensions: accuracy, completeness, consistency, timeliness, validity, and uniqueness. The frameworks also help the research team focus on potential errors in the data sets and to generate rules to address those errors. We use the term observation data because the primary goal for collecting the data from patients was for clinical care not for research purposes.

To facilitate the process of identifying and refining data errors in the depression registry, we used the Kahn framework. This framework consists of 5 components: (1) attribute domain constraints, (2) relational integrity rules, (3) historical data rules, (4) state-dependent object rules, and (5) attribute dependency rules. Using these components, we were able to measure the accuracy, validity, consistency, uniqueness, and completeness of data in the registry. Although evaluating the quality of data for the timeliness was out of the scope of this study, Table 1 shows the rules we generated for each component to identify and solve the data errors in the depression registry.

**Table 1:** Framework for analysis of data errors.

Dimensions of data quality	Components	Generated rules with examples
Accuracy, consistency, validity, and completeness	Attribute domain constraints	Define the domain of elements in the registry Use the frequency analysis to identify data elements with values out of the range of the domain Identify inconsistencies in the data elements’ domain Examples: All items in the PHQ-9^a^-questionnaire should have a value between 0-3. All answers to the question about the “current employment status” should be recorded as Yes or No. All other answers such as “I am employed” or “I am looking for a job” should be converted to Yes and No, respectively.
Consistency, uniqueness, and completeness	Relational integrity rules	Identify unique identifiers (primary keys) in each data set for mapping different data sets. Define appropriate strategies for mapping data sets. Example: The variables Medical-record-number, eligibility- date, and activation-date can be used as identifiers for mapping patients across different data sets.
Accuracy, consistency, validity, and completeness	Historical data rules	Identify data elements capturing date and time (date-time) of events in the IBH^b^ registry. Identify data elements that their differences present duration for an event (eg, duration of the CCM^c^). Identify date-time elements that needed to be recorded in a specific time interval. Examples: Date of birth should be recorded before all events associated with an individual patient in the registry. The difference between the activation-date (start date) and the end-date indicates the duration of the intervention. Each enrolled patient should have at least one contact date, otherwise the contact date should be labeled as a missing value.
Completeness, consistency, uniqueness, and accuracy	State-dependent objects rules	Identify a set of events whose occurrence depends on other events in the registry. Identify a set of events whose occurrence is concurrent with other events in the registry. Examples: Patients with an end date for the CCM intervention should also have a start-date and eligibility-date; otherwise, the start date and eligibility date would be labeled as missing values. The recording date for completing the PHQ-9, MDQ^d^, GAD7^e^, and AUDIT^f^ questionnaires should be before or at the same date of enrollment date.
Accuracy	Attribute dependency rules	Evaluate accuracy of events that follow other events. Identify data elements having aggregated values of associated components of the data elements. Examples: The total score of the PHQ-9 should be equal to the sum of the values of 9 items of the questionnaire.

^a^PHQ-9: Patient Health Questionnaire.

^b^IBH: Integrated Behavioral Health.

^c^CCM: care coordination model.

^d^MDQ: Mood Disorder Questionnaire.

^e^GAD7: General Anxiety Disorder-7.

^f^AUDIT: Alcohol Use Disorders Identification Test.

#### Addressing Missing Data in Patient-Centered Registries

Addressing the missing data in patient-centered registries depends on (1) frequency of missing data; (2) the source of missing data, which might be due to reasons such as patients’ unwillingness to share the requested information with clinicians, clinicians’ failure to collect the required information, data entry and processing error, and changing the guidelines of data collection of the registry; and (3) the type of missing data that can be summarized as missing completely at random (MCAR), missing at random, and missing not at random. See Multimedia Appendix 2 [24] for more information on the source and type of missing data. There are different approaches to handling the missing data with complete case analysis and imputation methods using machine learning or aggregation methods as common solutions.

**Figure 2:**
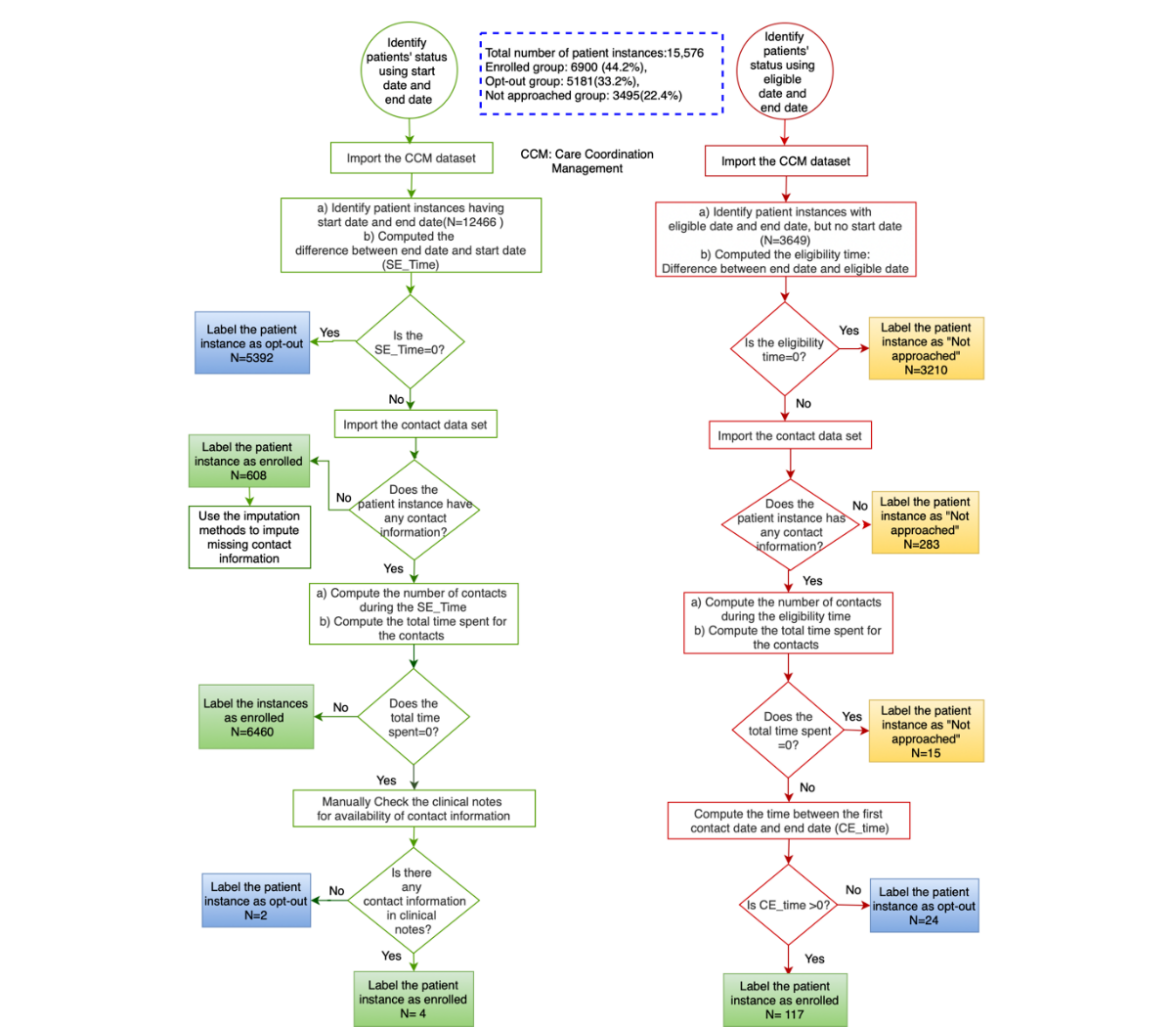
Identifying patient subgroups in the depression registry. CCM: collaborative care model.

The following are examples of our methods for handling missing values in the depression registry:

1. Using the complete case analysis for handling missing data in the CCM intervention data set: According to the guidelines of the depression registry, all patients enrolled in the CCM intervention should have an eligibility date, a start date, and an end date. Patient instances with a start date but without an end date were marked as missing data. As the number of missing data was low (0.4% or 90 out of a total of 18,716) and the source and type of the missing data were data entry error and MCAR, respectively, we simply dropped these patients from inclusion in the registry.
2. Developing an algorithm to identify and handle patient instances with a missing start date for the CCM intervention: To identify patient instances with a missing start date, we first computed the
  eligibility-time
  (end date minus eligibility date). If eligibility-time >0, we computed the total number of contacts and total time spent on communication between clinicians and patients during the eligibility-time. For patient instances with contact information, the first date of contact was marked as the start date. Overall, we identified 154 patient instances with a missing start date for the CCM intervention. Figure 2 visualizes the steps of this algorithm.

### Identifying Patient Cohorts in Patient-Centered Registries

To conduct comparative effectiveness research and health outcome studies using longitudinal data collected for clinical purposes, the research team needs to identify comparable patient cohorts and to define proper observational and outcome windows before entering the phase of data modeling and analysis. This is more complicated in registry data collected over long periods of time, as patients may have a wide variety of interactions recorded. In this section, we discuss the challenges of identifying patient cohorts and defining outcome windows in patient-centered registries, and then, we provide an approach to address these challenges based on the depression registry.

#### Excluding Ineligible Patients Based on the Specifications of the Research Question

Commonly, the first step in defining patient cohorts in registries for a specific research question is to define inclusion and exclusion criteria related to the aims of the study. For example, using the data in the depression registry, we were interested in investigating the effectiveness of the CCM intervention in improving health care outcomes in patients with depression. To answer this research question, we started with the inclusion and exclusion criteria, which, as illustrated in the DFD (Figure 1), was defined as all adult patients (≥18 years) who had a clinical diagnosis of major depression, with no prior diagnosis of bipolar disorder and with moderate to severe symptoms of depression (PHQ ≥10) before or on the start date of the enrollment for the CCM intervention. Patients who did not meet these criteria were excluded from the study sample.

#### Identifying Major Patient Subgroups in Patient-Centered Registries

The second step in defining patient cohorts in a patient registry is to identify major patient subgroups. The DFD of the registry can be very helpful in providing information about the different patient statuses (eg, enrolled or opt out), which can provide an insight into the possible patient subgroups in the database. Consultation with clinicians can also be helpful in identifying the subgroups.

In our case study, the DFD (Figure 1) shows that an individual patient may experience 3 possible statuses in the depression registry: enrolled, opt out, and not approached. After a discussion with the research clinicians, we utilized 2 data sets, the CCM data set and the contact data set to design 2 algorithms (algorithms A and B in Figure 2) to identify the 3 patient statuses in the registry. The CCM data set recorded the eligibility date of the patients for the intervention of CCM (eligible date), the date that a patient was recruited by a care coordinator (start date), and the date that the intervention ended (end date). All patient instances in the CCM data set were required to have an eligible date and end date, but the start date could be missing if the patient was not approached by clinicians for the CCM intervention. We computed 2 variables, activation-time (SE_Time) and eligibility-time using this data set, indicating the time that the patient status was open in the CCM intervention and in the registry, respectively. The contact data set included the number of contacts that occurred between care coordinators and patients for the CCM intervention and the associated time spent for each communication. Using these data sets, we computed the frequency and time of contacts (total time spent) between care coordinators and patients if the patients’ activation-time or eligibility-time was greater than 0. If the frequency=0 or total-time-spent=0, the patient was considered not enrolled in the CCM intervention. Table 2 presents the description, counts, and min/max distribution of the key date variables in the CCM and contact data sets used in algorithms A and B (Figure 2) for identifying major patient subgroups as well as some derived variables.

**Table 2:** Descriptions of and statistics of variables used in algorithms A and B (Figure 2).

Variables		Description	No.	No missing	Minimum	Maximum
**CCM^a^ database**						
	Eligibility date	The date that the patient was eligible for the CCM intervention.	15,576 (patient instances)	0	March 3, 2008	May 17, 2018
	Start date	The date that the care coordinator offered the CCM to the patient.	15,576 (patient instances)	3649 (23.42); patient instances having missing start-date	March 6, 2008	May 14, 2018
	End date	The end date of the CCM intervention.	15,576 (patient instances)	0	March 3, 2008	May 15, 2018
**Computed variables using the CCM database**						
	Activation-time (SE_Time)	Difference between the start date and the end date.	11,927 (76.57); patient instances having start-date	0	0 (days)	1263 (days)
	Eligibility-time	Difference between the eligibility date and the end date, if the start date is missing.	3649 (23.42) patient instances having eligibility date but without start date	0	0 (days)	900 (days)
**Contact database**						
	Contact date (date of contact)	The date of contact between the patient and the care coordinator.	121,435 instances of contact	206 (0.17)	March 7, 2008	September 28, 2018
	Time spent	The time spent for each contact.	121,435 instances of contact	0	0	990 min
**Computed contact variables for patient instances with activation-time >0**						
	Total number of contacts for each patient instance (algorithm A)	Total number of contacts between the clinician and the patient with activation-time >0.	6824; total number of enrolled patient instances having contact information	90 (1.32); total number of patient instances with no contact information	1 (number of contact)	123 (number of contact)
	Total time spent for each patient instance (algorithm A)	The total time that the clinician spent for communicating with the patients with activation-time >0.	6824; total number of enrolled patient instances having contact information	90 (1.32); total number of enrolled patient instances with no contact information	0 (min)	3375 (min)
**Computed contact variables for patient instances with eligibility-time >0**						
	Total number of contacts for each patient instance (algorithm B)	Total number of contacts between clinicians and patients with eligibility-time >0.	433; total number of patient instances with eligibility time >0	263 (60.73); total number of patient instances with eligibility time >0 and having no contact information	1 (number of contacts)	27 (number of contacts)
	Total time spent for each patient instance (algorithm B)	The total time that the clinician spent for communicating with patients with eligibility-time >0.	433; total number of patient instances with eligibility time >0	263 (60.73); total number of patient instances with eligibility time >0 and having no contact information	0 (min)	645 (min)
	CE_Time (algorithm B)	Duration between the first contact date (in the contact data set) and the end date (in the CCM data set).	154 (35.56); total number of patient instance s having at least one contact date	0	0	616

^a^CCM: care coordination model.

#### Identifying Observation and Outcome Windows for Patients With Multiple Statuses in the IBH Registry

**Figure 3:**
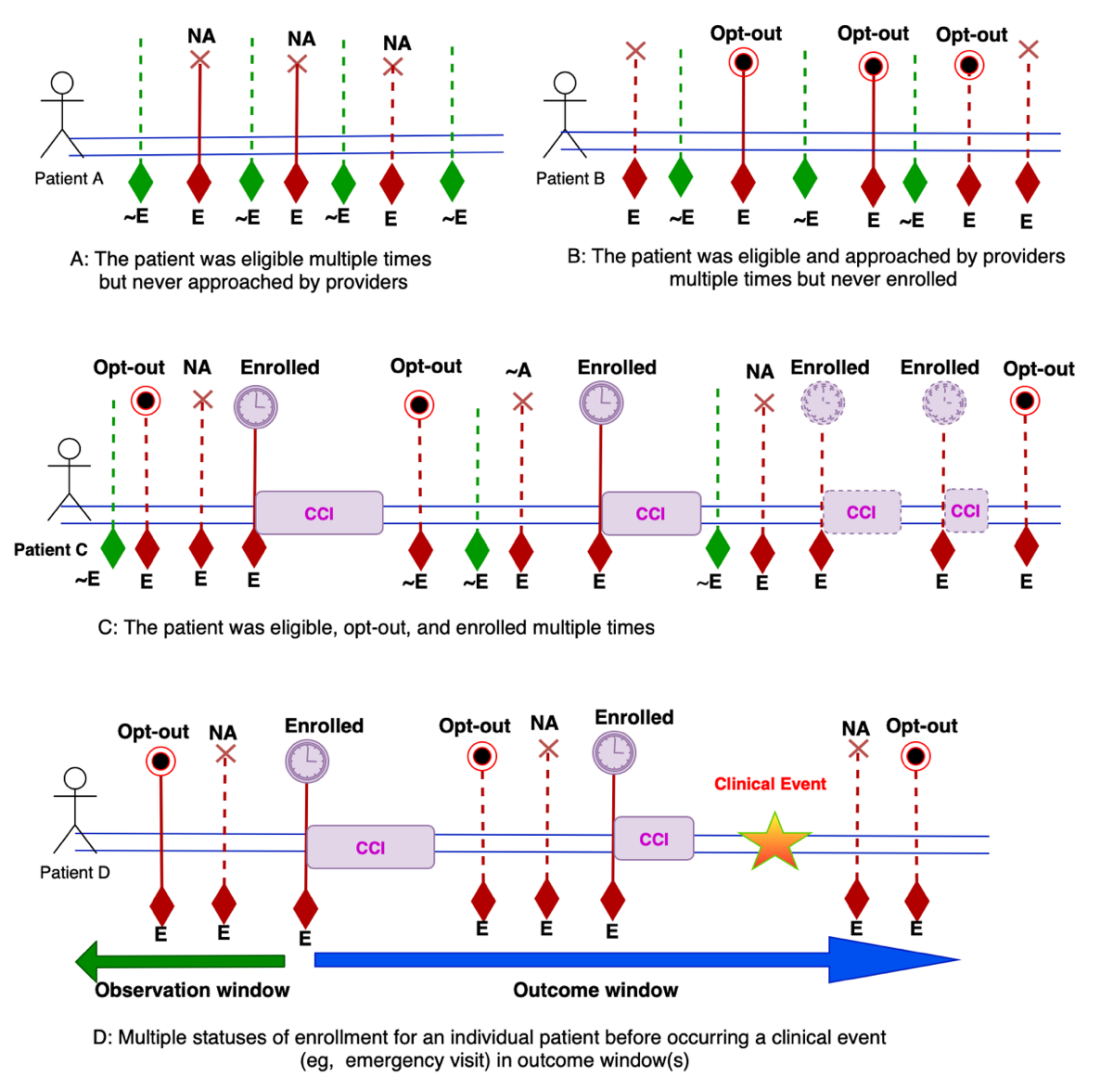
An illustration of a patient with multiple statuses in the Integrated Behavioral Health registry. CCI: care coordination intervention; ~E: not eligible; E: eligible; NA: not approached.

The third step for defining patient cohorts in a patient registry for a specific research question is to identify proper observation and outcome windows of patients with chronic illnesses who are followed over several years and have multiple points of eligibility due to the fluctuating nature of their condition. In chronic disease research where patients are followed over time, choosing observation and outcome windows can be difficult, primarily due to the heterogeneous behavior of the patients and the clinical program. For example, in the depression registry, a patient may be eligible multiple times for the intervention but never approached (nonapproached patients) by providers to enroll in the CCM intervention (part A in Figure 3). On the other hand, an eligible patient may be approached by the provider multiple times but refused to enroll in the intervention (opt-out patient, part B in Figure 3). However, some eligible patients may enroll in the intervention multiple times (part C in Figure 3). It is possible for multiple variations to occur with the patient not approached for one occurrence, opt out for another, and then enroll for another.

If the focus of the research question is on measuring the effectiveness of an intervention for a specific outcome such as an emergency department visit or rehospitalization, it is often unclear how to optimally define an index date (start of follow-up) for the 3 subgroups (enrolled, opt out, and not approached) identified in Section *Patient Registry DFD* and presented in Figure 3 parts A, B, and C. For example, for patients with multiple enrolled statuses, the index date can be defined as the start date of the first enrollment or it could be the start of a later enrollment. Each of the selected index dates may not necessarily lead to the same conclusion about the effectiveness of the intervention. Changes in practice (eg, the introduction of a new antidepressant or clinical service) may happen over time, which could impact clinical outcomes for patients starting CCM during a given period versus another one. Similarly, for patients with multiple opt-out statuses and who have never enrolled, their index date can be taken as the first, the last, or an interim opt-out date. It is not clear which is optimal; the different index dates may lead to different conclusions. In complex situations, setting an arbitrary index date to define observation and outcome windows could increase the risk of measurement bias (part D of Figure 3).

Our solution for identifying a proper index date to define patient cohorts for a cross-sectional study focused on measuring the coverage and effectiveness of the intervention for a specified follow-up time window (eg, 6 months) using the depression registry data. Thus, identifying a proper index date for a longitudinal study with a focus on health care outcomes would be part of our future study.

#### Identifying Patient Cohorts for a Cross-Sectional Study in the Depression Registry

To identify patient cohorts for a cross-sectional study, the focus was on measuring the effectiveness of CCM interventions in treating eligible registry patients with moderate to severe symptoms of depression. The depression database contains a PHQ-9 table containing all the questionnaires completed by those primary care patients who were treated at Mayo Clinic since 2008 and who completed at least one PHQ-9 in the course of their care. Patients were selected for the study if they were diagnosed with depression and met the inclusion/exclusion criteria (Figure 1) and completed at least two PHQ-9 questionnaires. Patients not meeting these criteria were excluded from the study.

For 6-month follow-up, there were 3 possible ways of specifying an appropriate index date for defining outcomes. In the first option, the index date would be the same for the entire cohort (intervention and comparison groups) and would be the first date on which a patient met the eligibility criteria. The advantage of this method was that all patients had a similar window of comparison. The disadvantage was that the time between the eligibility and enrollment dates may vary, such that the treatment effect was diluted with patients in the intervention group by being enrolled late in the observation window. The second option was to use an index date that was linked with enrollment into the CCM program. For the treatment group, this would be an intent-to-treat group where all enrolled patients were included regardless of completing or dropping out or reaching remission in treatment. For the comparison (usual care) group, however, the choice of an index date would be challenging. An eligibility date could be chosen, but if there were several, which one to choose? An additional concern is how to account for variation in the time between eligibility and enrollment in the treatment group as compared to the nontreatment group where (by definition) there was no enrollment. In the treatment arm, there is a possibility that patients began receiving some sort of treatment outside of the CCM program between their eligibility date and enrollment, which might bias results in favor of the treatment arm. The third option would be to look at the average time between eligibility and enrollment in the treatment cohort and add that time to each of the comparison cohort’s eligibility date to create an equivalent 6-month window.

**Figure 4:**
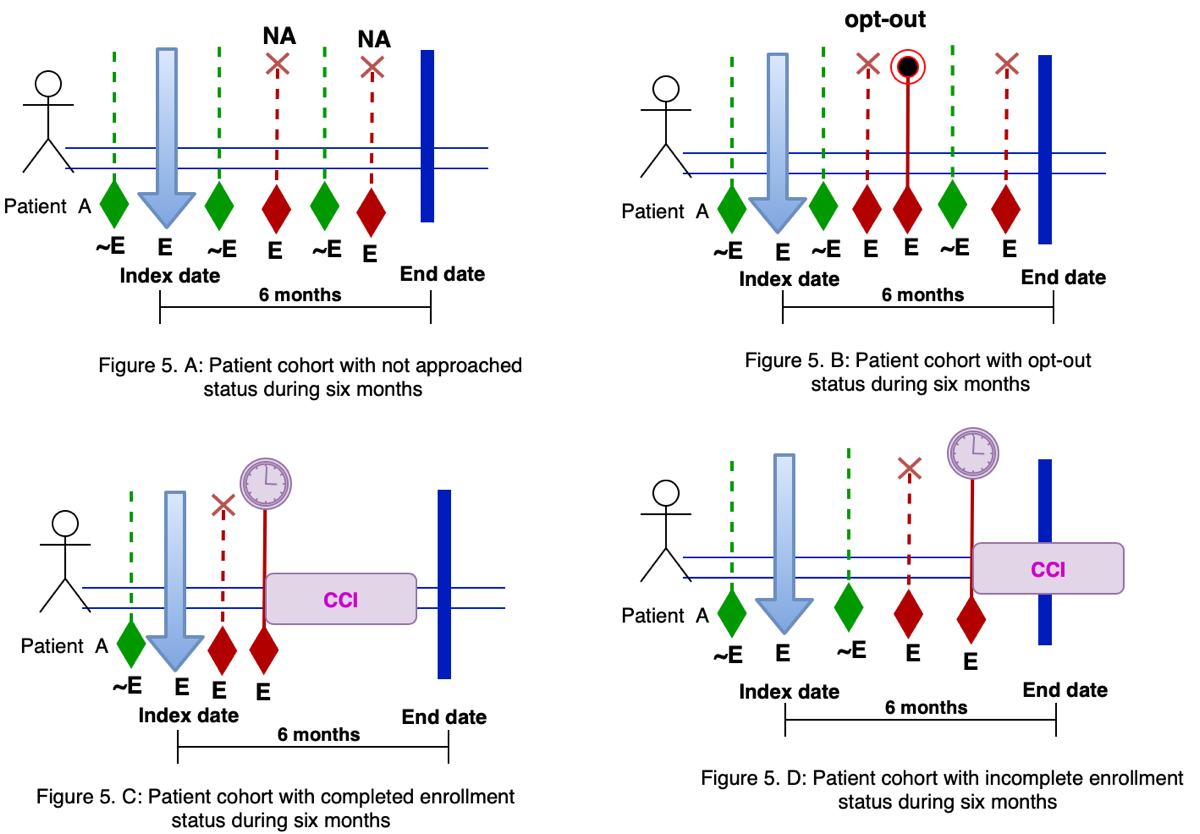
The identified patient cohorts for a cross-sectional study. CCI: care coordination intervention; E: eligible; ~E: not eligible; NA: not approached.

Choosing any of the index dates would result in generating different patient cohorts. In the special case where the index date was the date where the patient first became eligible, we identified 4 patient cohorts in the depression registry presented in Figure 4. Figure 4 parts A-D illustrates the patient cohorts with *not approached* status, *opt-out* status, *completed enrollment* status, and *incomplete enrollment* status at the 6-month window. Using these 4 cohorts, we aimed to test the following hypotheses in our next study:

1. Effectiveness will be higher in patients with enrolled status (vs others) as measured by the percentage of patients reaching remission in 6 or 12 months and/or time to remission. Those patients who choose to enroll in care coordination are different (by age, etc) than those who either are not approached or opt out, suggesting a need for changes in the program or in recruitment efforts to better impact the population of depressed patients. There is a significant difference between demographic information (age, employment, education, and marital status) and comorbidities in patients with *enrolled* status compared with patients without *enrolled* status (*not approached* and *opt-out* status).
2. There is a significant difference between the frequency of emergency department visits and hospitalizations in patients with *enrolled* status compared with patients with *not approached* and *opt-out* status in the outcome window of 12 months after the index date (the 6 months after the index date).

## Results

### Summary of the Components of the Analytical Framework

The primary result of this project was the development of a clean data set with descriptors of the data and patient cohorts for a research project. Table 3 summarizes the main steps in taking a clinically created patient-centered registries and getting the data ready for research.

**Table 3:** A summary of the analytical framework suggested in this study.

Problem	Solution	Advantages	Limitations	Other options
Patient care programs evolve based on clinical and reimbursement changes without regard to research leading to confusion about the flow of data for research	Create a data flow diagram	Provides insight into the underlying structure of data Identifies the main patient cohorts available in the registry	Not suitable for registries that are lack of the component of patient-centered care for chronic disease	Unified Modeling Language [25] Conceptual Modeling [26]
Data collected in clinical settings is prone to many data quality issues	Use the Kahn et al [23] framework to evaluate the quality of accumulated data in the registry against 5 dimensions: accuracy, completeness, consistency, validity, and uniqueness	Provides specific operational approaches to determine the quality of data in a patient-centered registry	Not appropriate for multisite registries Not appropriate for cleaning unstructured data set (eg, text cleaning)	Achilles Heel Data Quality Tool [27]
Patients may flow in and out of clinical care based on clinical needs leading to confusion when creating cohorts	Use visualization techniques to visualize all possible instances (having no, single, or multiple enrollment status) in the registry	Helps the research team define key points of time in a patient’s flow (eg, eligibility date and start date) that account for the majority of patients Helps create rule-based algorithms to create comparable patient cohorts for a study	Needs a deep understanding of patients’ flow in the registry and standard definitions that are adhered to in clinical practice as patients enter and leave treatment.	Use unsupervised machine learning algorithms (eg, deep learning) for creating initial patient cohorts for human review [28]

### A Descriptive Summary of Patients’ Demographic Information and PHQ-9 Data Available in the Depression Registry

The total number of patients registered in the depression registry was 18,716 patients as of 2008. Consent is sought and obtained annually by the clinical practice as a part of regular care and documented in the EHR for retrospective patient research at the Mayo Clinic. In this project, 7.01% (1312/18,716) of the registry patients (n=1310) did not consent to share their data for research purposes and were excluded from the study. Out of 17,406 patients with informed consent, about 1830 (10.51%) did not meet the inclusion criteria (a history of bipolar disorder, age 18 years or greater, or a PHQ-9<10) and were excluded from the study. Overall, out of 15,576 eligible patients, we identified 6900 (44.2%) instances with enrolled status, 5181(33.2%) with opt-out status, and 3495 (22.4%) with nonapproached status. Table 4 summarizes the key baseline characteristics of these subgroups. The mean age of the enrolled, opt out, and nonapproached subgroups was 41.3 (SD 16.2), 40.2 (SD 16.5), and 41.6 (SD 17.5), respectively. The proportion of females in all 3 subgroups was significantly higher than that of males, which is in line with the findings of the Centers for Disease Control and Prevention, indicating that two times as many women use antidepressants as men [29]. Whites comprised a large fraction (>90%) of patients in the 3 groups. Similarly, the majority of the patients in all 3 groups were married (enrolled: 3377/6900,48.94%; opt out: 44.85%, 2324/5181; and not approached: 1461/3495, 41.80%), followed by single patients (enrolled: 2102/6900,30.46%; opt out: 1841/5181,35.53%; and not approached: 1354/3495, 38.74%). Table 3 also includes the depression score as measured by the PHQ-9 questionnaire for the 3 subgroups. The majority of patients in all 3 groups had moderate depression (PHQ-9 between 10 and 19) at both the eligibility date and start date of the CCM intervention. Patients with severe depression (PHQ-9≥20) were more likely to be enrolled in the CCM intervention. In future studies, we will evaluate the association of demographic information and the PHQ score with patients’ willingness to accept or refuse to participate in the CCM intervention.

**Table 4:** Statistics on major patient subgroups in the depression registry.

Variables		Enrolled	Opt out	Not approached
Total number of instances, n (%)		6900 (44.30)	5181 (33.30)	3495 (22.40)
Age (years), mean (SD)		41.27 (16.18)	40.17 (16.46)	41.59 (17.54)
**Sex, n (%)**				
	Female	4936 (71.50)	3758 (72.50)	2402 (68.70)
	Missing	10 (0.14)	6 (0.10)	1 (0.02)
**Race, n (%)**				
	White	6347 (91.98)	4675 (90.23)	3197 (91.47)
	Black or African American	157 (2.27)	152 (2.93)	91 (2.60)
	Asian	116 (1.68)	92 (1.77)	32 (0.91)
	Native American	28 (0.40)	26 (0.50)	28 (0.80)
	Others	188 (2.72)	184 (3.55)	109 (3.12)
	Unknown	55 (0.79)	45 (0.87)	37 (1.10)
	Missing	9 (0.13)	7 (0.13)	1 (0.02)
**Marital status, n (%)**				
	Married	3377 (48.94)	2324 (44.85)	1461 (41.8)
	Single	2102 (30.46)	1841 (35.53)	1354 (38.74)
	Divorced	1084 (15.71)	802 (15.48)	487 (13.93)
	Widowed	320 (4.63)	203(3.92)	177 (5.06)
	Unknown	7 (0.10)	4 (0.07)	15 (0.40)
	Missing	10 (0.14)	7 (0.13)	1 (0.2)
**PHQ-9^a^ at start-date, n (%)**				
	≤5^b^	69 (1.00)	153 (2.95)	No start date is available
	>5, <10^b^	260 (3.78)	134 (2.58)	No start date is available
	≥10, <15	2956 (42.84)	2401 (46.34)	No start date is available
	≥15, <20	2309 (33.46)	1593 (30.74)	No start date is available
	≥20 (severe depression)	1228 (17.79)	824 (15.90)	No start date is available
	Missing	78 (1.13)	76 (1.46)	No start date is available
**PHQ-9 at eligibility date, n (%)**				
	≤5^b^	17 (0.25)	0	0
	>5, <10^b^	49 (0.71)	0	0
	≥10, <15	3055 (44.27)	2537 (48.97)	1976 (56.54)
	≥15, <20	2491 (36.10)	1751 (33.80)	1011 (28.93)
	≥20 (severe depression)	1288 (18.67)	893 (12.23)	508 (14.53)

^a^PHQ-9: Patient Health Questionnaire.

^b^The enrolled patients with PHQ-9<10 at the point of enrollment were included in the data set of the study due to the health care providers’ discretion. The patients met other eligibility criteria and were diagnosed with depression. The clinical reasons for inclusion of these subthreshold patients were varied and included a previous pattern of relapse or a concern that the patient was minimizing their symptoms.

## Discussion

### Principal Findings

A well-designed and implemented registry that ensures comprehensive, consistent, accurate, and complete data about patients is critical for accurate assessment of disease burden, evaluation of disease management and health care services, and conducting comparative effectiveness and outcomes research. The success of retrospective research studies utilizing data from patient registries depends on the underlying structure of the registry, the implemented data preparation steps, and the research question(s). This study focused on the data preparation steps (DFD and data quality) to support statistical or machine learning data analysis applications and selection of patient cohorts to address specific research questions in mental health. The feasibility and usefulness of the proposed framework was demonstrated using the depression patient-centered registries at the Mayo Clinic, which was designed for the CCM [30] to manage primary care patients diagnosed with moderate to severe depression. By following the data cleaning and refining procedure discussed in this framework, we produced high-quality data for potential research questions that can be answered using the data in the depression registry. We also generated cohorts of patients available for testing hypotheses related to the effectiveness of the CCM for primary care patients with depression.

In patient registries developed to follow patients with chronic diseases over multiple years, identifying appropriate patient cohorts for cross-sectional or longitudinal studies can be very challenging. In the case of the CCM, a patient can have multiple points of enrollment for the intervention due to the fluctuating nature of chronic conditions. A potential solution, as discussed in this study, is to first identify major patient subgroups (eg, enrolled, opt out, not approached) in the registry and set the index date as the first date of eligibility for all subgroups and limit the outcome window to a relatively short period (eg, 6 months) after the index date. Although this solution can help select the right patients (intervention and control) for a cross-sectional study, it may not be appropriate for longitudinal studies because some patients may have multiple eligibility and enrollment statuses. One possible solution would be to use multiple outcome windows (eg, every 6 months after the index date). In this case, information collected in a previous outcome window can be included as patient history to assess the effectiveness of the intervention in the next outcome window.

An additional area of consideration for practices embarking on adopting or creating a patient-centered registry in their setting would be to reduce the potential challenges we identified upstream of the point at which research is done. Data collected in clinical practice as compared with data collected in research settings are complicated by who does the data entry and changes in staff. Tools such as the EHR and patient workflows evolve and can lead to a lack of oversight. Data may be gathered to report quality outcomes or to support billing, but it is often rare to see practices that ensure data quality for quality improvement and retrospective research. Practices might consider creating a DFD during patient-centered registries design, along with standard definitions of critical elements (eg, eligibility vs start date and graduation vs recovery). Monitoring data integrity and assigning oversight of the registry, highlighting the importance of data maintenance, would vastly reduce the time involved in preparing clinical data for research and allow for more rapid feedback to the practice about which programs are or are not effective.

### Lessons Learned

A summary of lessons learned during the process of developing this systematic framework and testing its feasibility using data in the depression registry was as follows:

1. Data cleaning and refining of accumulated data in a patient-centered registry is a time-consuming process and unexpectedly challenging. Researchers need to plan to dedicate sufficient time and resources to understand the underlying structure of the data and develop effective procedures to identify and manage data quality issues in the registry.
2. Visualizing multiple statuses of an individual patient over a period of time would highlight the challenges of defining appropriate observation and outcome windows for this group of patients. Therefore, it would help the research team to adopt a proper strategy for defining appropriate patient cohorts and consequently reduce the risk of measurement bias in the study.
3. Involving stakeholders of the patient registry (eg, care coordinators and primary care providers) in the process of addressing data quality issues, specifically missing data, would substantially assist the research team in adopting appropriate strategies for handling the issues and consequently would provide high-quality data for subsequent research projects.

### Limitations

We acknowledge some limitations of our proposed framework:

1. The first component of data analysis is mostly suitable for designing a DFD in registries with a focus on patient-centered interventions for managing chronic diseases. Therefore, the DFD might not be appropriate for other types of health care registries with a different focus or patient group.
2. Data quality has many different dimensions. In this study, we discussed 5 dimensions of data quality: accuracy, completeness, consistency, timeliness, validity, and uniqueness. Information about other dimensions such as timeliness or accessibility can be found in the study by Kodra et al [16].
3. The second component of the registry is mostly developed with a focus on cleaning and refining structured data. If unstructured data (eg, clinical notes or images) are also part of the analyses, then the research team needs to implement the applicable methods for the unstructured data.
4. Registries vary in their strengths with regard to being linked to pharmacy data, administrative data, and care in multiple sites. Some of these methods would vary as data from other sources are included.

### Conclusions

There is a need for a conceptual and methodological framework for generating and evaluating data-driven hypotheses using data in patient-centered registries created for clinical reasons to enhance care and outcomes for patients. The systematic framework introduced in this study provides a clear step-by-step process for identifying and managing data quality issues in the registries and identifying appropriate patient cohorts for a specific research question. Overall, it is unrealistic to aim for data in a registry that is completely free of errors. Some errors will remain undetected and uncorrected regardless of the completeness of the data quality assessment framework. Utilization of a data analytics framework can merely lead to an improvement in data quality. We selected the components of data quality and identified patient cohorts in this framework to be practically feasible and facilitate detecting and correcting common errors in the registries. This implies that this framework can be expected to be effective in providing high-quality data to evaluate data-driven hypotheses using data in patient-centered registries. In future studies, we aim to use the data in the depression registry to estimate the risk of hospitalization and emergency department visits, measuring the effectiveness of the CCM on clinical outcomes and health care services cost.

## Appendix

Multimedia Appendix 1 Integrated Behavioral Health registry.

Multimedia Appendix 2 Missing data.

## Abbreviations

CCM: collaborative care model
DFD: data flow diagram
EHRs: electronic health records
IBH: Integrated Behavioral Health
MCAR: missing completely at random
PHQ-9: Patient Health Questionnaire
