# Supplementary material for: A Systematic Framework for Analyzing Observation Data in Patient-Centered Registries: Case Study for Patients With Depression
Source: JMIR Res Protoc. 2020 Oct 29;9(10):e18366. doi: 10.2196/18366 (PMC7661226; doi:10.2196/18366)
Supplement: Multimedia Appendix 1 [file resprot_v9i10e18366_app1.docx]

**Appendix A: IBH registry**

In response to the combined challenges of lack of access to care for depression and the need for a way to monitor clinical outcomes of a cohort of depressed adults in primary care , Mayo Clinic, division of Integrated Behavioral Health (IBH),Department of Psychology and Psychiatry initiated a patient registry to support the, “collaborative care model” (CCM), to deliver” integrated” and “coordinated” mental health and general healthcare services to diverse patients with depression in primary care setting (<https://www.cms.gov/Outreach-and-Education/Medicare-Learning-Network-MLN/MLNProducts/Downloads/BehavioralHealthIntegration.pdf>). The CCM is a patient-centered multicomponent intervention with key pillars includinga care coordinator, a registry, routine measurement of patient outcomes, and ongoing regular (weekly in our case) review of patient outcomes with a psychiatric provider leading to adjustments in the patient’s plan of care in order to ‘treat to target.’

The registry was envisioned to be used for a variety of purposes; tracking tasks needed in the care of patients for care coordinators, assisting in weekly review to identify which patients need more intervention or are ready to discharge, assisting administrators in providing real time data on patient outcomes for QI purposes and/or to compare outcomes at various sites to identify best practices, and to assist in answering a variety of retrospective research questions. As compared with pulling information from individual charts, the registry was a significant step forward.However, in regards to the potential for the registry, the challenges of keeping the registry functioning and linked to the patient medical record were formidable. This led to some choices such that certain aspects of the registry were more robust than others (tracking tasks for example). Any real-time link to administrative or pharmacy data was not possible, requiring such information to be pulled only for retrospective research. The registry thus has been more potent in making sure patients are not lost to follow up versus knowing which patients are trending into higher use of unnecessary care. In 2017, with the introduction of Collaborative Care Management (CoCM) codes by the Centers for Medicare and Medicaid Services, and as Mayo moved to a new electronic record, the registry functions were pulled into EPIC. This led to new challenges in data mapping for any longitudinal case review.

With the move to integrate the registry into EPIC, there is increased potential to link to data collected for other reasons (social determinants of health, medical comorbidities, use of higher cost services, etc). It remains to be seen if this potential can lead to reports in real time that allow treating providers to better recognize cohorts of patients who need further attention as identified in ways beyond their pHQ-9 or involvement with the care coordinator. The Components enable providers to systematically take into account the complex medical, psychological, social, and cultural factors in a patient’s treatment plan and consequently provide a personalized treatment regimen that enables patients with depression to reach remission in a shorter time with lower cost.

The depression registry includes information on diverse patients with respect to disease severity, treatment protocols, comorbidities, and socioeconomic and ethnic backgrounds. Additionally, it is equipped with the mechanism of data integration (such as data standardization and operability) which enables the providers and researchers to integrated the registry data with administrative, pharmacy, emergency and hospitalization databases, as well as patients’ social determinant of health and personal health records.
