# Supplementary material for: A Systematic Framework for Analyzing Observation Data in Patient-Centered Registries: Case Study for Patients With Depression
Source: JMIR Res Protoc. 2020 Oct 29;9(10):e18366. doi: 10.2196/18366 (PMC7661226; doi:10.2196/18366)
Supplement: Multimedia Appendix 2 [file resprot_v9i10e18366_app2.docx]

**Appendix B: Missing Data**

In clinical data, identifying and addressing missing data is a common challenge. Adopting appropriate strategies for addressing missing values can reduce the risk of introducing bias to the findings of the study and enhance quality of the research. Briefly, addressing the missing values in patients’ registries depends on 1) frequency of missing values, 2) source of missing values, and 3) type of missing values. In patient centered registries, the source of missing values can be summarized as patients’ unwillingness to share the requested information with providers, failure to collect required information from patients, and data entry and processing error. Additionally, changing the guidelines of data collection of the registry, such as adding new data elements or changing the status of data elements from optional to required ones would result in missing data for those elements.

The type of missing values can be summarized in three classes of randomness: 1) missing completely at random (MCAR); 2) missing at random (MAR); and 3) missing not at random (MNAR). Missing data having the MCAR type are independent of observed and unobserved data elements (variables) in the registry system. For instance, in the depression registry, the type of missing data for “activation-date” and “end-date” for the intervention is the MCAR, and the source of the missing data is providers’ mistakes. In contrast to the MCAR, missing data with the MAR type are dependent on the observed variables in the dataset. For example, if the total number of PHQ-9 dataset depends on the collected variables in the IBH registry (e.g., age, gender, education, or the patient’s history of diagnosis), the type of the missing data is MAR. On the other hand, missing data classified as MNAR are systematically related to the variables that were not measured by clinicians or researchers (unobserved variables). To extend pervious example, if the total score of PHQ-9 data depends on the unavailable information in the dataset, such as the patient’ life style component, the type of missing value is MNAR.

Methods for handling missing data can be classified into two broad categories: 1) complete case analysis (CCA) strategy, and 2) imputation methods. In complete case analysis, the patient instances with missing values would be excluded from the analysis. This strategy is useful when the frequency of missing data is relatively small, and the type of missing data is MCAR. However, when the frequency of the missing data is relatively high or type of missing data is MAR or MNAR, using the CCA strategy would create bias to the findings and reduce the power of the study. Unlike the CCA, the focus of the imputation methods is on replacing the missing data with a single value (or multiple values). Aggregation methods (e.g., mean or median) or machine learning (ML) approaches such as random forest algorithm or clustering methods could be used for imputing missing data. The ML algorithms focus on identifying the most similar patient instances with complete data to the instances with missing data based on a set of predefined covariant, thus they are more effective than aggregation methods. However, the effectiveness and validity of the algorithms would be affected by the sample size, complexity of the patients’ illness, and the type of missing data. Another common way for imputing missing data in longitudinal observational dataset is the Last observation carried forward (LOCF) or the next observation carried backward (NOCB). The LOCF or NOCB are useful when the patient is required to complete a specific medical test in each follow up visit (e.g., PHQ-9 questionnaire in depressed patients). In this case, the missing date for the test in a visit could be imputed by the value of the test in other visits. See [30] for more information about imputation methods.
